# The P2X7 Receptor Promotes Intestinal Fibrosis by Modulating the Gut Microbiota and the Inflammasome

**DOI:** 10.1016/j.jcmgh.2025.101718

**Published:** 2025-12-30

**Authors:** Beatriz Elias Ribeiro, Isadora Schmukler de Lima, Karen Cristina da Silva e Souza, Siane Lopes Bittencourt Rosas, Patrícia Teixeira Santana, Gilda Amaral, João Carlos Machado, Rodrigo Pereira de Oliveira, Camille Leal, Cristiane Thompson, Fabiano Thompson, Morgana Teixeira Lima Castelo-Branco, Robson Coutinho-Silva, Heitor Siffert Pereira de Souza

**Affiliations:** 1 Department of Clinical Medicine, Federal University of Rio de Janeiro, Rio de Janeiro, Brazil; 2 D’Or Institute for Research and Education (IDOR), Rio de Janeiro, Brazil; 3 Biomedical Engineering Program, COPPE, Federal University of Rio de Janeiro, Rio de Janeiro, Brazil; 4 Institute of Biology, Federal University of Rio de Janeiro, Rio de Janeiro, Brazil; 5 Institute of Biomedical Sciences, Federal University of Rio de Janeiro, Rio de Janeiro, Brazil; 6 Institute of Biophysics Carlos Chagas Filho, Federal University of Rio de Janeiro, Rio de Janeiro, Brazil

**Keywords:** Fibrogenesis, Gut Microbiota, Inflammasome, Inflammatory Bowel Disease, P2X7, Purinergic Signaling

## Abstract

**Background & Aims:**

Considering the role of the P2X7 receptor in intestinal inflammation, we examined its potential involvement in fibrosis development.

**Methods:**

Colonic biopsies from patients with inflammatory bowel disease (IBD) were analyzed via double immunofluorescence under confocal microscopy. Colon fibroblasts were used to analyze P2X7 receptor modulation and chemotaxis. Experimental chronic colitis was induced with 3 cycles of oral dextran sodium sulfate (DSS) treatment in P2X7^+/+^ and P2X7^-/-^ mice. The mice were evaluated via follow-up video endoscopy with an endoluminal ultrasound biomicroscopic (eUBM) system, histological scoring, immunohistochemistry, cytokine measurement in colon explants, gene expression analysis of P2X7 signaling targets via quantitative real-time polymerase chain reaction (qRT-PCR), and microbiome composition analysis.

**Results:**

Colocalization studies revealed a greater density of P2X7^+^/alpha smooth muscle actin (α-SMA)^+^ cells in colon sections from patients than in those from controls, especially in patients with Crohn’s disease (*P* < .05). Activation of the adenosine triphosphate (ATP)-P2X7 pathway in human fibroblasts increased cell migration, calcium influx, and collagen production. Video colonoscopy with the eUBM system revealed significantly more inflammation, with greater wall thickness and stiffness, in P2X7^+/+^ mice than in P2X7^-/-^ and P2X7^+/+^ mice treated with A740003 (a P2X7-selective inhibitor). P2X7^+/+^ mice exhibited increased caspase-1 and NLRP3 expression, as well as nuclear factor κB (NF-κB) and extracellular signal-regulated kinase (ERK) activation, accompanied by decreased peroxisome proliferator-activated receptor gamma (PPARγ) expression. In the supernatants of colon explants, tumor necrosis factor (TNF)-α, interleukin (IL)-1β, interferon (IFN)-γ, transforming growth factor (TGF)-β, IL-10, and collagen production were increased in P2X7^+/+^ mice. Various microbial changes were observed in P2X7^-/-^ and P2X7^+/+^ mice.

**Conclusions:**

Regulatory mechanisms downstream of P2X7, combined with signals from a dysbiotic microbiota, activate intracellular signaling pathways and the inflammasome, leading to intestinal inflammation and promoting fibrogenesis.

## Graphical abstract

**Figure:**
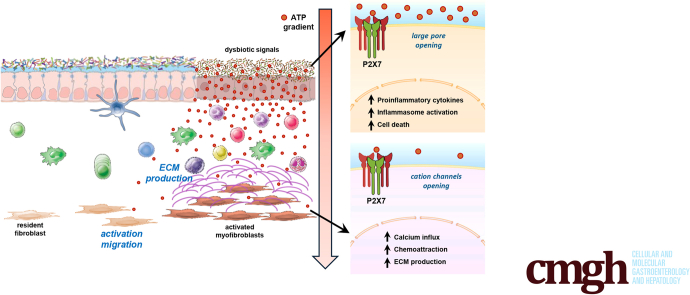

**Summary**

The P2X7 receptor is overexpressed in the chronically inflamed colon and colocalizes with lamina propria myofibroblasts significantly more in human inflammatory bowel disease samples than in noninflamed control samples. The experimental model confirms the role of the P2X7 receptor in amplifying and sustaining the inflammatory process, as well as inducing a fibrotic response, in mice with chronic colitis.

**What You Need to Know**

**Background**

The P2X7 receptor is overexpressed in the chronically inflamed intestine of patients with inflammatory bowel disease, and exposure to adenosine triphosphate maintains a local inflammatory environment that promotes fibrosis development.

**Impact**

The resultant fibrogenesis is driven by regulatory mechanisms downstream of P2X7, including calcium influx, fibroblast migration, enhanced extracellular matrix synthesis, and the inflammasome activation, combined with signals from a dysbiotic microbiota.

**Future directions**

Targeting P2X7 in inflammatory bowel disease is postulated as a novel approach for potentially inhibiting excessive fibrogenesis.

Intestinal fibrosis may develop as a late outcome for different chronic gastrointestinal disorders, including Crohn’s disease (CD) and ulcerative colitis (UC), collectively referred to as inflammatory bowel disease (IBD).^1^ A potentially severe complication of IBD, particularly CD, is intestinal fibrosis, which occurs in more than one-third of patients,^2^ resulting in luminal narrowing and frequently requiring surgical treatment.^3^ Currently, the available therapies for IBD are immunosuppressants and biological medications aimed at inducing remission of inflammation. Nevertheless, no treatment prevents or reverses established tissue fibrosis.^4^

During the fibrotic process, activated myofibroblasts expand from other mesenchymal-like cells, undergo transcellular differentiation, and migrate toward the sites of injury, controlled by gradients of autocrine and paracrine molecules, such as transforming growth factor (TGF)β1, reactive oxygen species (ROS), and peroxisome proliferator-activated receptor (PPAR), among others.^5^ In the intestinal milieu, mucosal myofibroblasts were shown to be activated by microbiota-derived molecules through interactions with pattern recognition receptors such as Toll-like receptors (TLRs).^6^ Nevertheless, other independent molecular elements from noninflammatory pathways are also involved in fibrogenesis. Among them, damage-associated molecular patterns (DAMPs), which are diverse non-microbial-derived molecules released from tissue injury, can act as signaling mediators, amplifying the inflammatory response and independently activating myofibroblasts.^7^

In addition to fragments of extracellular matrix (ECM) components, free nucleic acids, and microvesicles, adenosine triphosphate (ATP) released in the extracellular milieu due to tissue injury secondary to infection or cellular stress is also considered a DAMP that can regulate various physiological cell functions upon its ligation with membrane purinergic receptors, such as P2X7.^8^ However, prolonged exposure of the P2X7 receptor to ATP can induce transmembrane potassium and calcium flux, initiating intracellular signaling pathways that lead to the downstream activation of the NLRP3 inflammasome^9^ and the recruitment of caspase-1. These changes result in the release of active interleukin (IL)-1β,^10^ a proinflammatory cytokine that promotes the release of TGF-β1, a central profibrotic mediator.^11^ Through this mechanism, the P2X7 receptor has been shown to play a role in the development of chronic inflammation and fibrosis in various organs, including the lungs, kidneys, liver, pancreas, and heart.^12^

Previous studies from our group have revealed that P2X7 receptor expression is increased in inflamed areas of the intestinal mucosa in both experimental and human IBD. In various models of experimental IBD, we have demonstrated the proinflammatory effects of the P2X7 receptor in both acute^13^ and chronic^14^ conditions, thereby amplifying and sustaining inflammation.^15^ Nonetheless, the full development of chronic intestinal inflammation has been associated with signals from dysbiotic gut microbiota, directly or indirectly modulated by the ATP-P2X7 pathway.^14^^,^^16^ In turn, the reduced microbial diversity, specific microbial imbalances, and altered metabolite production characterizing gut dysbiosis in IBD have also been associated with the presence of pro-fibrotic microbial-associated molecular patterns (MAMPs).^17^ However, the potential role of the ATP-P2X7 pathway in intestinal fibrosis remains unclear. Given the above-mentioned evidence, we hypothesized that purinergic signaling through the P2X7 receptor could also be modulated in mesenchymal cells, regulating chronic intestinal inflammation and, consequently, fibrosis development. Therefore, in this study, we investigated the expression and function of the P2X7 receptor in intestinal fibroblasts and the potential therapeutic effect of P2X7 receptor blockade in an experimental model of intestinal fibrosis.

## Results

### P2X7 Expression in Mesenchymal Cells of the Colonic Mucosa of Patients With IBD

Serial colon sections were stained with hematoxylin and eosin (H&E) to assess inflammatory activity, and phosphomolybdic acid-picrosirius red was used to evaluate collagen fibers.^18^ At least 5 areas per tissue section were analyzed by light microscopy using a computer-assisted image analyzer. Paraffin sections were also used to characterize P2X7 and PPAR-γ expression in the colons of patients with CD, UC, and normal controls. After antigen retrieval, the sections were analyzed by indirect immunoperoxidase using monoclonal mouse anti-P2X7 or anti-PPAR-γ antibodies, along with appropriate controls, including an isotype-matched monoclonal immunoglobulin G (IgG) at the same concentration.

**Figure 1:**
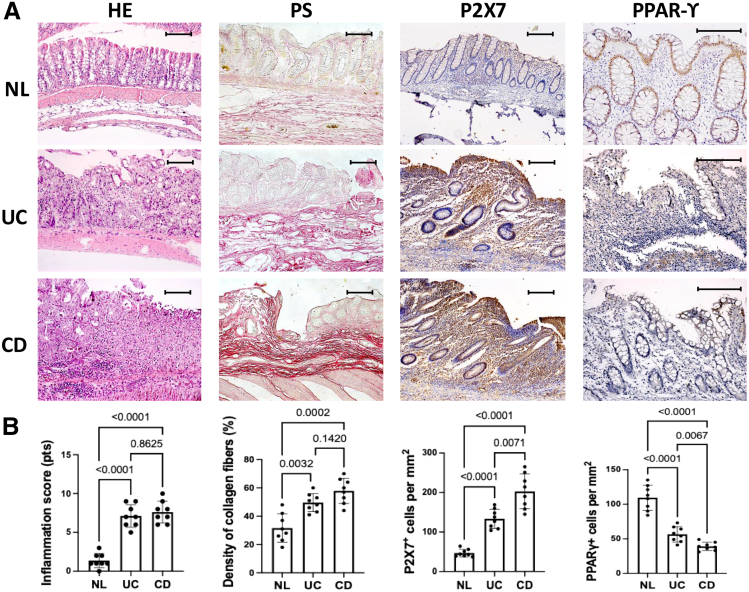
**Compared with controls (NLs), patients with CD and UC showed significantly greater inflammatory indices (HE) and collagen fiber densities (PS).** P2X7 and PPAR-γ, which are differentially expressed in the colon, were analyzed by immunoperoxidase staining, which revealed diffuse staining of P2X7 in the lamina propria and epithelial cells of the inflamed colon. In contrast, in the normal noninflamed areas, only faint staining was observed in both compartments. PPAR-γ staining revealed predominant epithelial expression in NLs, whereas its expression was universally reduced in inflamed areas. The scale bars represent 100 μm (*A*). Individual values are presented, along with their means and standard deviations. Significant differences between the experimental groups were identified using Welch’s ANOVA test, followed by multiple comparisons with Dunnett’s T3 test, as appropriate (*B*).

Regarding histological grading of inflammation, 5 specimens from macroscopically inflamed colons of patients with CD were classified as severe, and 3 as moderate. Among the samples obtained from patients with UC, 5 were classified as severe and 3 as moderate. All mucosal biopsies from the control group were histologically normal. In patients with IBD, the inflammatory changes were accompanied by increased collagen fiber deposition and enhanced P2X7 receptor expression. In contrast, PPAR-γ expression was significantly reduced by inflammation. These findings demonstrate that chronic inflammatory changes, including fibrosis, are topographically associated with the overexpression of P2X7 receptor and the concomitant underexpression of PPAR-γ (Figure 1).

Next, we investigated the differential expression of the P2X7 receptor in mesenchymal cells of the colonic mucosa from patients using double immunofluorescence staining and confocal analysis. To identify which mesenchymal cells coexpressed the P2X7 receptor, we incubated sections with anti-P2X7 (red), anti-SMA (green), or anti-vimentin (green). Colocalization was defined as the number of double-positive cells (yellowish color) relative to the single-positive cells in the lamina propria per mm^2^ (counted in at least 4 different areas). Co-expression was further confirmed by confocal laser scanning microscopy using the software's colocalization analysis tool. Briefly, images were captured at distinct excitation wavelengths (488 nm and 633 nm for Alexa-conjugated fluorophores), creating excitation-lambda stacks. Using an acousto-optical beam splitter, excitation lambda stacks were separated into individual images that correspond to signals from different dyes, eliminating autofluorescence and crosstalk between fluorochromes. Two independent observers, unaware of the patients’ or experimental data, examined all tissue sections and captured images.

**Figure 2:**
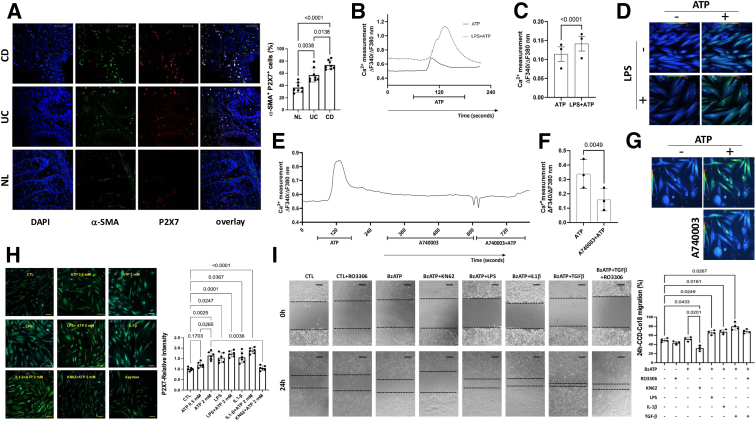
**The colocalization of P2X7 (*red*) with α-SMA (*green*) was significantly greater in the samples from the patients with CD and UC than in those from the noninflamed controls (NL).** (*A*). Nuclei are stained with DAPI (*blue*). The micrograph panels are representative of 8 control, 8 CD, and 8 UC mucosal samples (original magnification, ×1000). The scale bars represent 50 μm. The percentages of double-positive cells in the colonic lamina propria are individually represented (*A*). Intracellular calcium ([Ca^2+^] i) levels were assessed in CCD-18Co cells after plating at 1 × 10^5^ cells per well on 15 mm coverslips. Upon exposure to ATP, a transient rise in calcium is observed, returning to basal levels after ∼2 minutes. However, pre-incubation with LPS enhanced the calcium influx peak, followed by a slower return to baseline after ∼3 minutes (*B* and *C*). The micrograph panel is representative of 3 experiments for each condition (*D*). Next, pretreatment with the P2X7 receptor antagonist A740003 strongly inhibited the calcium influx observed in ATP-incubated cells (*E* and *F*). The micrograph panel is representative of 3 experiments for each condition (*G*). In another set of experiments, the relative intensity of P2X7 in the CCD-18Co cells subjected to different treatments was determined via confocal microscopy. Compared with the controls, P2X7 expression significantly increased after treatment with ATP (0.5 mM or 2 mM), LPS, IL-1b, with or without ATP (2 mM). In contrast, expression decreased after KN62 + ATP (2 mM) compared with LPS + ATP (2 mM). The micrograph panel is representative of 7 experiments for each condition. The scale bars represent 20 mm (*H*). The effects of different stimuli that activate P2X7 receptors on CCD-18Co migration were analyzed by light microscopy. Panels show images of migration from 0 to 24 hours into scratch sites following growth in medium containing vehicle or other stimuli. Compared with the KN62-treated cells, the BzATP-treated cells previously exposed to LPS, IL-1β, or TGF-β exhibited significantly increased migration. The scale bars represent 20 μm. The micrograph panel is representative of 4 experiments for each condition (*I*). Individual values are presented, along with their means and SDs. For comparing the means of 3 or more groups, the analyses were performed using Welch’s ANOVA test, followed by multiple comparisons using Dunnett’s T3 test. Paired samples *t*-test was used to determine differences in calcium influx experiments. Significant values are highlighted.

In the inflamed CD lamina propria, P2X7 receptor colocalized predominantly with alpha smooth muscle actin (α-SMA)-positive cells (median, 71.0%) and to a lesser extent with vimentin-positive cells (median, 23.5%). In CD, the inflamed mucosa expressed proportionally more P2X7 receptor in the α-SMA-positive cells than the control (*P* = .001) and UC (*P* = .0462) mucosa (Figure 2*A*). The proportion of P2X7-positive, vimentin-positive cells did not differ significantly among the groups (not shown). The overexpression of the P2X7 receptor on α-SMA-positive cells in the inflamed lamina propria suggests that the ATP-P2X7 pathway may play a role in the activation and function of these cells, potentially also affecting fibrogenesis.

### Effect of the ATP-P2X7 Pathway on Intracellular Calcium in Human Colon Fibroblasts

Changes in intracellular calcium [Ca^2+^] levels were used to evaluate P2X7R function in CCD-18Co human colon fibroblasts. The variations in Ca^2+^ were measured by quantifying the fluorescence ratio emitted by Fura-2 after alternating excitation at 340 and 380 nm. Images captured at different time points were processed and analyzed in groups of 20 to 40 cells. Treating colon fibroblasts with ATP transiently increased intracellular calcium, which returned to baseline after approximately 2 minutes. However, pre-incubation with lipopolysaccharide (LPS) (1 μM for 24 hours and 1 hour before calcium measurements) significantly increased the peak of the calcium influx curve, which then returned to baseline after more than 3 minutes (Figure 2*B* and *C*). A representative micrograph panel showing cells exposed to ATP and/or LPS is shown (Figure 2*D*). Next, we examined whether A740003 could inhibit P2X7R activation. Pretreatment with A740003 significantly reduced the calcium influx triggered by ATP in colon fibroblasts (Figure 2*E* and *F*). A representative micrograph panel showing fibroblasts exposed to ATP and treated with the P2X7R antagonist A740003 is shown (Figure 2*G*).

### Expression and Modulation of P2X7 in Human Fibroblasts

We used CCD-18Co colon fibroblasts to investigate P2X7 receptor expression and modulation, and to analyze P2X7 receptor protein levels in response to various stimuli, including ATP (0.5 mM and 2 mM), LPS, and IL-1β. Confocal microscopy of immunofluorescence-stained fibroblasts revealed a significant increase in basal P2X7 receptor levels in cultures upon stimulation with ATP 2 mM, LPS, and IL-1β. This change was prevented by pretreatment with KN62, a selective inhibitor of the human P2X7 receptor (Figure 2*H*).

### The ATP-P2X7 Pathway Affects Fibroblast Chemotaxis but Not Cell Proliferation or Viability

The ability of the ATP-P2X7 pathway to affect human fibroblast chemotaxis, cell proliferation, viability, and death was investigated using a scratch-wound assay, 5-Bromo-2′-deoxyuridine (BrdU) incorporation quantification, the 3-(4,5-dimethylthiazol-2-yl)-2,5-diphenyltetrazolium bromide (MTT) assay, and caspase-3 activity, respectively. Concerning chemotaxis, BzATP alone failed to induce the migration of CCD-18Co colon fibroblasts. However, a notable difference was observed between BzATP-treated and KN62-treated cells (*P* = .024). Significant differences were observed in combined treatment with BzATP and LPS, IL-1β, or TGF-β compared with untreated control cells (*P* = .0049, .0054, and .0041, respectively). To differentiate between cell migration and proliferation, we pretreated cells with RO3306, a Cdk1 inhibitor, to induce cell cycle arrest and prevent cell division (Figure 2*I*). These results suggest that ATP induces fibroblast chemotaxis upon pretreatment with inflammatory (IL-1β), microbial (LPS via TLR4), or growth factor (TGF-β)-related stimuli.

**Figure 3:**
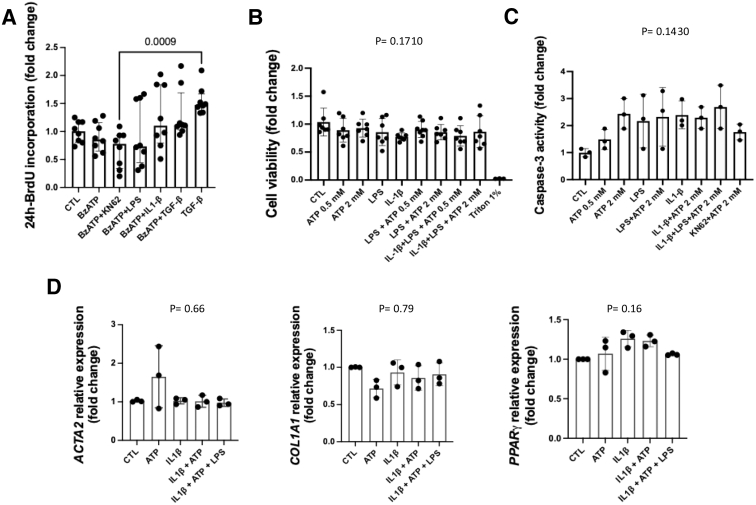
**The effect of the ATP-P2X7 pathway was assessed by measuring HFF1 proliferation, viability, caspase-3 activity, and fibrosis-related gene expression.** When cells were exposed to 0.5 mM or 2 mM ATP, LPS, and IL-1β, cell proliferation measured by BrDU incorporation (*A*); cell viability, as analyzed by the MTT assay (*B*); and the caspase-3 activity did not change significantly at 24 hours (*C*), all at 24 hours. Expression and modulation of mRNA for *ACTA2*, *COL1A1*, and *PPAR-γ* in HFF1 cells was determined by RT-qPCR. HFF1 cells were treated with ATP (0.5 mM or 2 mM), LPS, IL-1β, and KN62, with or without ATP (2 mM), and DMSO (vehicle) for 24 hours. Gene expression, showing fold changes for individual values, is normalized to the ββ-actin (*ACTB*) gene (*D*). Individual values are presented, along with their means and SDs. Significant differences between the experimental groups were identified with Welch’s ANOVA test.

Although ATP alone did not induce BrdU incorporation, the levels tended to increase when ATP was combined with LPS, IL-1β, or TGF-β. However, the KN62-treated HFF1 cells incorporated significantly less BrdU than the TGF-β-treated cells (*P* = .0009) (Figure 3*A*). Regarding fibroblasts’ viability, we analyzed HFF1 cells after exposure to ATP (0.5 or 2 mM), LPS, and IL-1β for 24 hours; the MTT assay did not show significant differences among the treatments (Figure 3*B*). Similarly, caspase-3 activity in HFF1 cells did not change significantly at 24 hours following exposure to ATP at 0.5 mM or 2 mM, LPS, or IL-1β (Figure 3*C*). Regarding mRNA for *ACTA2* (a-SMA), *COL1A1*, and *PPAR-γ* in HFF1 cells, none changed significantly at 24 hours following exposure to ATP at 0.5 mM or 2 mM, LPS, or IL-1β (Figure 3*D*).

### The ATP-P2X7 Pathway Increases Morbidity and Mortality and Promotes Inflammation and Fibrogenesis in a Mouse Model of Chronic Colitis

**Figure 4:**
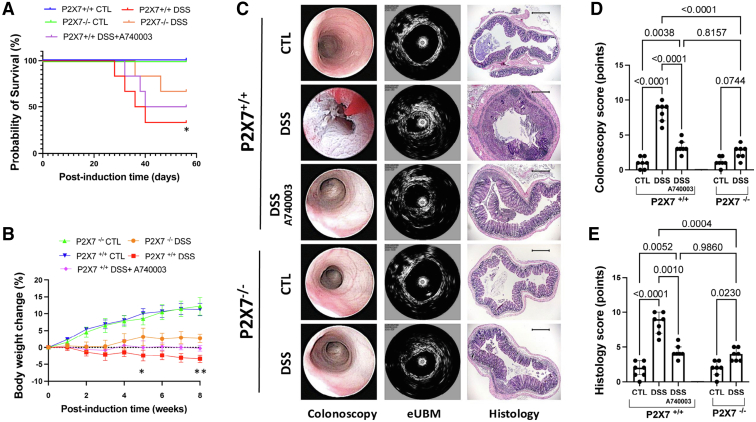
**At the end of week 8, a significant increase in mortality was observed in the DSS-induced P2X7R^+/+^ mice compared with the non-exposed control mice (*A*).** Compared with those in the control groups, the weight of the mice in the DSS groups did not increase over time (*B*). After the third DSS induction week, colonoscopy images were obtained 2 cm from the anus, and eUBM and histological axial analysis were performed (*C*). The colonoscopy (*D*) and the histological (*E*) scores were higher in the DSS-induced P2X7^+/+^ than in the P2X7^+/+^ control (CTL) group, the P2X7^-/-^ mice, and the DSS-induced P2X7^+/+^ treated with A740003. The analysis was performed using Welch’s ANOVA, with multiple comparisons conducted using Dunnett’s T3 test. ∗*P* < .05; ∗∗*P* < .01; ∗∗∗*P* < .001. Individual values are shown, along with the means and SDs from 2 independent experiments (5–7 per group).

To further investigate the potential role of the ATP-P2X7 pathway in chronic intestinal inflammation and fibrosis, we employed an experimental model of chemically induced chronic colitis in mice using dextran sulfate sodium (DSS). As shown in Figure 4*A*, the DSS-treated P2X7^+/+^ mice had higher mortality rates than the control P2X7^+/+^ mice did (*P* = .0236), whereas A740003 treatment attenuated the effect of DSS in P2X7^+/+^ mice. Body weight significantly decreased at the end of week 4 (day 28) in the DSS-induced P2X7^+/+^ mice compared with the control P2X7^+/+^ mice (*P* = .0197). Upon treatment termination at the end of week 8 (day 56), body weight changed even more in the DSS-induced P2X7^+/+^ mice than in the control P2X7^+/+^ mice (*P* = .0031), whereas A740003 treatment attenuated the effect of DSS in P2X7^+/+^ mice (Figure 4*B*).

To monitor the progression of intestinal fibrosis in vivo, we periodically performed video colonoscopy on the mice at various time points after DSS administration. As in previous works from our group,^14^ colonoscopy was performed with endoluminal ultrasound biomicroscopic (eUBM), a device that uses a high-frequency ultrasound system to acquire detailed cross-sectional images. After the first DSS cycle (third week), most animals showed mucosal edema and an abnormal vascular pattern. Ultrasonic images revealed a standard wall thickness and a clear distinction between the hyperechoic and hypoechoic layers. After the second cycle of DSS treatment (sixth week), all P2X7^+/+^ wild-type mice displayed mucosal granularity and friability, and most of them also had ulcers and bleeding. In contrast, the P2X7^+/+^ wild-type mice treated with A740003 did not present all the abnormalities observed in the untreated animals, whereas almost no change was detected among the DSS-induced P2X7^−/−^ animals. After the third cycle of DSS treatment (week 8), the colonic abnormalities were strongly exacerbated in the DSS-induced P2X7^+/+^ animals. The colonic mucosa showed advanced inflammatory changes and narrowing of the intestinal lumen. The cross-sectional images confirmed wall thickening in the hyper- and hypoechoic layers, with no clear distinction between them. Some induced wild-type mice treated with A740003 presented mucosal edema and vascular changes in the colonic mucosa; however, the well-defined layers under the ultrasonic images were preserved, indicating superficial changes only. Most P2X7^−/−^ mice exhibited a normal colon throughout the entire experiment; however, in some cases, mild inflammatory changes were observed at the end of the protocol. Notably, no ulcers or narrowing were detected (Figure 4*C* and *D*).

The histological assessment confirmed significantly increased inflammation and wall thickness in DSS-induced P2X7^+/+^ mice compared with P2X7^+/+^ control mice. However, only mild inflammatory changes were observed in the other DSS-induced animals, with no increase in wall thickness, as shown by the cross-sectional analysis (Figure 4*E*). These data suggest that the ATP-P2X7 pathway is crucial for the development of inflammation and fibrosis in DSS-induced chronic colitis.

### The ATP-P2X7 Pathway Promotes Colon Wall Stiffness and Increases the Secretion of ECM Components

**Figure 5:**
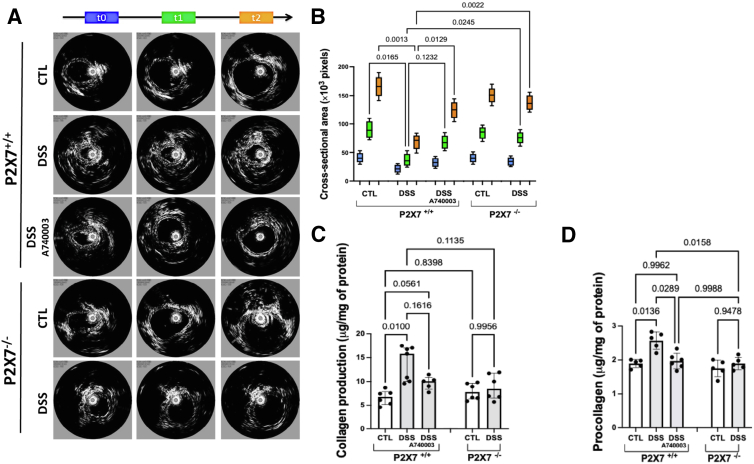
**eUBM was performed on anesthetized mice after the third week of DSS induction.** Colon stiffness was assessed with an intrarectal injection of 0.5 mL of saline solution at times t0, t1, and t2 (10 seconds in total) during ultrasound image acquisition (*A*). The increase in the transverse colon section area was analyzed in the experimental groups. The DSS-induced P2X7^+/+^ mice presented a lower increase in the transversal section area than the control, A740003-treated DSS-induced P2X7^+/+^, and DSS-induced P2X7^-/-^ mice (*B*). The analysis was performed using Wilcoxon’s signed-rank test. The horizontal bars represent the medians, and the boxes represent the 25th and 75th percentiles. The data are representative of 2 independent experiments (4–5 per group). Collagen production in colon homogenates from the P2X7^-/-^ and P2X7^+/+^ explants treated with A740003 was lower than in the DSS-induced P2X7^+/+^ explants (*C*). Procollagen production in colon homogenates from the P2X7^-/-^ and P2X7^+/+^ explants treated with A740003 was lower than in the DSS-induced P2X7^+/+^ explants (*D*). The analysis was performed using Welch’s ANOVA, with multiple comparisons conducted using Dunnett’s T3 test. Individual values are presented, along with the means and SDs of 2 independent experiments (5–7 per group).

Although experimental models of colitis cannot fully replicate fibrogenesis in IBD, we developed a new method for analyzing intestinal fibrosis in vivo, using a system that provides a simultaneous tridimensional view of the colon during colonoscopy. The eUBM system provides cross-sectional images and enables real-time dynamic evaluation of intestinal stiffness. In addition to increased wall thickness, DSS-induced P2X7^+/+^ mice showed significantly lower distensibility at both time points compared with the other groups. Notably, the damaging effects of DSS were significantly reduced in P2X7^+/+^ wild-type mice treated with A740003 (Figure 5*A* and *B*).

The increased collagen deposition, characterized by an imbalance between collagen synthesis and degradation, with excessive ECM deposition, underlies the thickening and stiffening of the intestinal wall. Therefore, in this study, we examined collagen and procollagen production from intestinal explants to assess the activation state of ECM-producing myofibroblasts within the chronically inflamed mucosa. The assessment of collagen secretion from intestinal explants revealed that the concentrations were significantly higher in the DSS-induced P2X7^+/+^ mice than in the P2X7^+/+^ control mice. In contrast, collagen production in P2X7^-/-^ mice did not change significantly after DSS exposure, and levels were similar to those in P2X7^+/+^ control mice (Figure 5*C*). Similar to the results obtained with collagen, the assessment of procollagen secretion from explants also showed increased levels in the DSS-induced P2X7^+/+^ mice. Nevertheless, besides the low levels obtained from P2X7^-/-^ explants, the chemical blockade with A740003 significantly prevented DSS-induced procollagen secretion by P2X7^+/+^ mice (Figure 5*D*).

### The ATP-P2X7 Pathway Promotes the Deposition of Collagen Fibers and Glycosaminoglycans in the Colon and Regulates Epithelial Integrity

**Figure 6:**
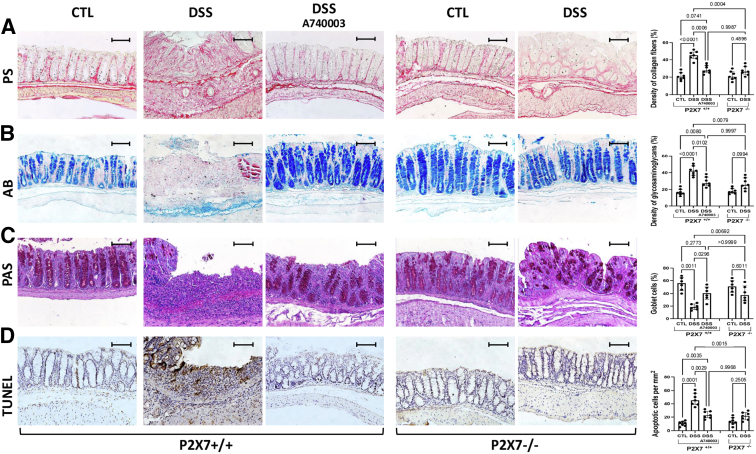
**In paraffin sections, ECM deposition and epithelial damage were analyzed in the DSS-induced chronic colitis model.** Picrosirius red (PS)-stained collagen fibers (*A*) and alcian blue (AB)-stained glycosaminoglycans were increased in DSS-induced P2X7^+/+^ mice compared with A74003-treated DSS-induced P2X7^+/+^ and P2X7^-/-^ mice (*B*). PAS-stained goblet cells were decreased (*C*), whereas the apoptotic rate, as assessed by a TUNEL assay, was increased (*D*) in DSS-induced P2X7^+/+^ mice compared with A74003-treated DSS-induced P2X7^+/+^ and P2X7^-/-^ mice. The analysis was performed using Welch’s ANOVA, with multiple comparisons conducted using Dunnett’s T3 test. Individual values are presented, along with the means and SDs of 2 independent experiments (5–7 per group).

The density of collagen fibers (Figure 6*A*) and glycosaminoglycans (Figure 6*B*) in colon tissue was significantly increased in the DSS-induced P2X7^+/+^ mice compared with the P2X7^+/+^ control mice. Although tissue sections from DSS-induced P2X7^+/+^ mice have a greater accumulation of collagen fibers and glycosaminoglycans, the absence of significant statistical differences compared with the other groups may be due to the relatively small sample size.

To assess epithelial integrity in colon samples, we stained goblet cells with Periodic Acid-Schiff (PAS) and analyzed cell death using a TdT-mediated dUTP-biotin nick end labeling (TUNEL) assay. Colon samples from inflamed areas of DSS-induced P2X7^+/+^ mice showed significantly lower percentages of PAS-positive cells than those from the control group (Figure 6*C*). Colon samples from inflamed areas of the DSS-induced P2X7^+/+^ mice presented significantly greater apoptosis rates than those from the control mice (Figure 6*D*).

Although the labeling of DSS-induced P2X7^+/+^ mice treated with A740003 did not differ, the results suggested a tendency toward reduced ATP-P2X7 pathway activation. Nevertheless, genetic ablation of the P2X7 receptor completely abrogated the effects of DSS induction on collagen deposition and accumulation of ECM glycosaminoglycans, as well as on epithelial barrier damage, as reflected in goblet cell numbers and apoptotic rates.

### The ATP-P2X7 Pathway Regulates the Production of Inflammatory Cytokines in the Colon

**Figure 7:**
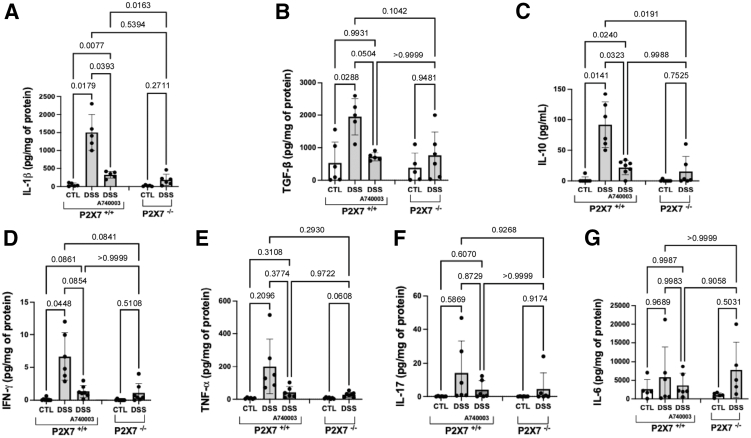
**Cytokine levels were assessed in the supernatants of colon explants cultured for 24 hours.** Genetic inactivation of P2X7 resulted in decreased levels of IL-1β (*A*), TGF-β (*B*), IL-10 (*C*), IFN-γ (*D*), TNF-α (*E*), IL-17 (*F*), and IL-6 (*G*) compared with those in P2X7^+/+^ mice. A740003 was used as a selective inhibitor of P2X7 for treating DSS-induced P2X7^+/+^ mice. The analysis was performed using Welch’s ANOVA test, followed by multiple comparisons using Dunnett’s T3 test. Individual values are presented, along with the means and SDs of 2 independent experiments (n = 5 per group).

Next, we investigated whether P2X7 affects the production of inflammatory mediators that may contribute to the inflammatory and fibrotic changes associated with the chronic model. To achieve this aim, we used a mouse Th1/Th2/Th17 cytokine kit based on bead-array technology to simultaneously detect interferon (IFN)-γ, tumor necrosis factor (TNF)-α, IL-2, IL-4, IL-6, IL-10, and IL-17A. TGF-β and IL-1β were analyzed separately via enzyme-linked immunosorbent assays (ELISAs). Analysis of supernatants from colon explant cultures revealed significantly increased concentrations of the proinflammatory cytokines TNF-α, IFN-γ, and IL-1β, as well as the anti-inflammatory cytokines TGF-β and IL-10, in samples from P2X7^+/+^ DSS-induced mice compared with controls. On the other hand, no significant change was detected between control and DSS-induced P2X7^-/-^ mice. The differential expression of these cytokines appears to corroborate the idea that chronic inflammation is a protracted process in which inflammation, tissue injury, and repair mechanisms coexist. Only the quantifiable results were analyzed (Figure 7).

### The ATP-P2X7 Pathway Modulates the Expression of IL-1β and NOS2 in Chronic Colitis

**Figure 8:**
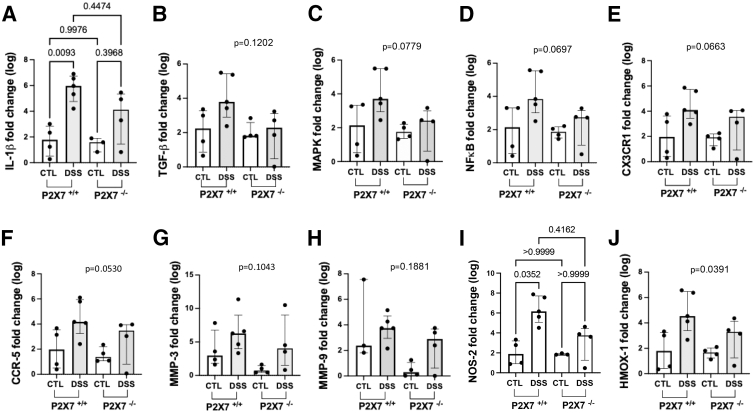
**P2X7 genetic inactivation altered the expression of genes related to inflammation and fibrosis in the experimental chronic colitis model.** The mRNA levels of IL-1β (*A*), TGF-β (*B*), MAPK (*C*), NF-κB (*D*), CX3CR1 (*E*), CCR-5 (*F*), MMP-3 (*G*), MMP-9 (*H*), NOS-2 (*I*), and HMOX-1 (*J*) were measured via qRT-PCR of colon samples. Individual values are presented, along with their means and SDs. Significant differences between the experimental groups were identified with Welch’s ANOVA test, in which multiple comparisons were carried out via Dunnett’s T3 test, as appropriate.

To investigate the mechanisms by which DSS regulates the inflammatory response and fibrotic changes in the colon, we examined mRNA expression of genes involved in tissue remodeling and inflammation. Although the results revealed a tendency toward upregulation of all target genes studied in P2X7^+/+^ DSS-induced mice, significant changes were observed only in IL-1β and nitric oxide synthase 2, inducible (NOS2) expression compared with controls. In the DSS-induced mice, IL-1β mRNA expression was also significantly greater in the P2X7^+/+^ mice than in the P2X7^-/-^ mice. No significant change in mRNA expression was detected between control and DSS-induced P2X7^-/-^ mice (Figure 8). These findings appear to corroborate the key role of the ATP-P2X7 pathway in IL-1β production and its positive correlation with increased NOS2 expression observed during prolonged inflammatory challenges. Nevertheless, the results from some of the other genes analyzed in this study could have been affected by the relatively small number of samples and the specific time point of the transversal analysis, which may not reflect the dynamic changes that occurred during the development of chronic inflammation.

### The ATP-P2X7 Pathway Regulates the Activation of Intracellular Pathways and the Inflammasome in Chronic Colitis

**Figure 9:**
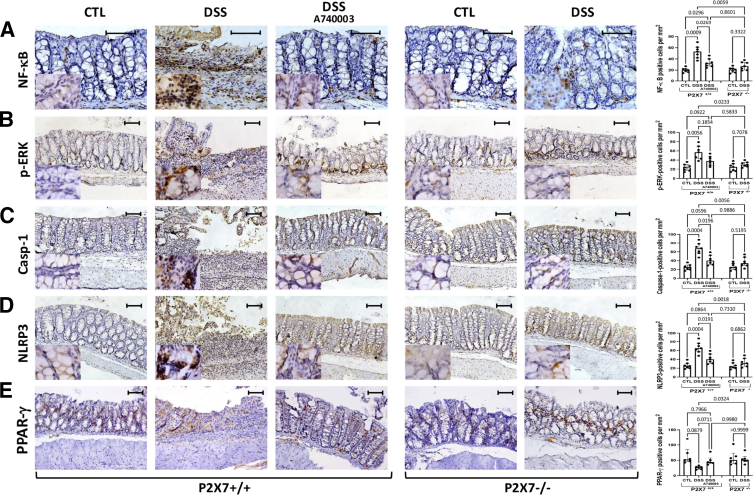
**Paraffin sections of colon samples stained with indirect immunoperoxidase were analyzed via a computerized image analysis system.** P2X7 receptor blockade via genetic inactivation significantly attenuated the increase in the number of NF-κB (*A*), phosphorylated ERK (*B*), caspase-1 (*C*), and NLRP-3 (*D*) -positive cells observed in DSS-induced P2X7^+/+^ mice. Using the oil-immersion technique, most positive cells for NF-κB, p-ERK, and caspase-1 in slides from DSS-induced P2X7^+/+^ mice showed intense nuclear staining, whereas in the other groups, staining was markedly weaker and mostly cytosolic (*A–C*). For NLRP3, positive cells in slides from DSS-induced P2X7^+/+^ mice showed intense, diffuse cytosolic staining, whereas in the other groups, staining was markedly weaker (*D*). In contrast, P2X7 receptor blockade decreased the number of PPAR-γ-positive cells (*E*). A740003 was used as a selective inhibitor of P2X7 for treating DSS-induced P2X7^+/+^ mice. The scale bars represent 50 μm. The analysis was performed using Welch’s ANOVA, with multiple comparisons conducted using Dunnett’s T3 test. Individual values are shown, along with the means and SDs of 3 independent experiments (n = 7 per group).

To confirm the findings from cytokine measurements, we also investigated the intracellular signaling pathway and inflammasome activation at the protein level by immunohistochemistry. Muclear factor κB (NF-κB), phosphorylated (p) extracellular signal-regulated kinase (ERK) mitogen-activated protein kinase (MAPK), caspase-1, and NLRP3 displayed similar expression patterns and tissue distributions. NF-κB, p-ERK, caspase-1, and NLRP3 were present in the epithelium and lamina propria at significantly greater densities in the inflamed areas of the P2X7^+/+^ DSS-induced mice than in those of the control mice. Using the oil-immersion technique to increase magnification, we achieved finer resolution and brightness in the slides, thereby highlighting the subcellular localization of the proteins under investigation. In NF-κB, p-ERK, and caspase-1, most positive cells in slides from DSS-induced P2X7^+/+^ mice showed intense nuclear staining, whereas in the other groups, staining was markedly weaker and mostly cytosolic (Figure 9*A–C*). Regarding NLRP3, positive cells in slides from DSS-induced P2X7^+/+^ mice showed intense, diffuse cytosolic staining, whereas staining in the other groups was markedly weaker (Figure 9*D*). In contrast, P2X7 receptor blockade decreased the number of PPAR-γ-positive cells. Significantly lower PPAR-γ expression was detected in inflamed areas of the DSS-induced P2X7R^+/+^ mice than in those of the DSS-induced P2X7R^-/-^ and control mice. No significant change was detected between control and DSS-induced P2X7^-/-^ mice (Figure 9*E*).

### ATP-P2X7 Modulates the Microbiota Associated With Inflammation and Fibrosis in Chronic Colitis

**Figure 10:**
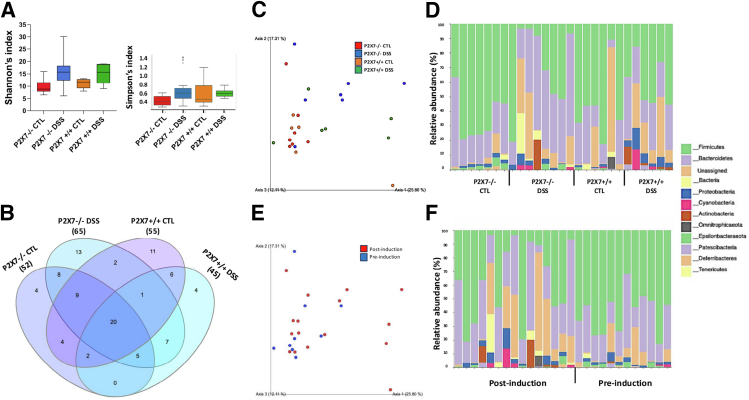
**Alpha-diversity analysis, using the Shannon and Simpson indices, revealed a downward trend in fecal microbial diversity when P2X7 was blocked (*A*).** Beta diversity analysis via weighted UniFrac PCoA clustered DSS-induced samples from control samples (*C*) and samples before and after induction (*D*). A Venn diagram displays the logical relationships among groups (*B*). Differential abundance analysis of the taxonomic profiles revealed the microbial composition at the phylum level (*E* and *F*). Sequencing was performed with samples from 2 independent experiments, with 6 to 8 animals per group.

**Figure 11:**
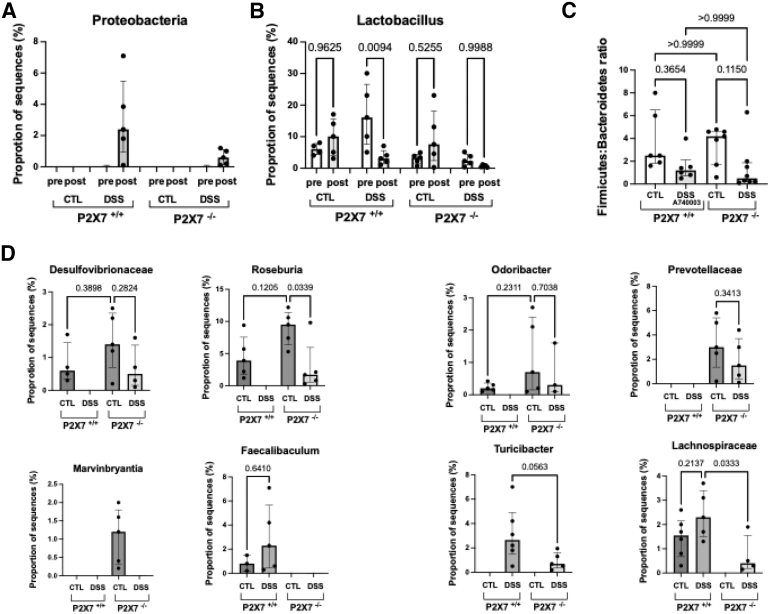
**Effect of P2X7 blockade on the microbiota composition in fecal samples from the experimental chronic colitis model.** The relative abundance of OTUs is shown for the phylum Proteobacteria (*A*), the genus *Lactobacillus* (*B*), the ratio of Firmicutes to Bacteroidetes (*C*), and at different levels of microbial classification hierarchy (%) (*D*). The values are the medians with interquartile ranges of 5 animals per group. The analysis was performed via the Kruskal-Wallis test, in which multiple comparisons were carried out via Dunn’s post hoc test. Significant values are presented.

To determine whether the abundance of specific bacteria is associated with the degree of fibrosis, we performed 16S rRNA sequencing to characterize the fecal microbiome. We then investigated the potential association between the differentially abundant microbes and the experimental groups. High-throughput sequencing produced 118,478 16S rRNA sequences (300 nt in length) after deblur quality control clean-up of 27 samples (P2X7^+/+^ control 28,579 ± 2779; P2X7^+/+^ DSS 20,763 ± 1562; P2X7^−/−^ control 16,848 ± 575; P2X7^−/−^ DSS 52,288 ± 3823; values represent the mean ± standard deviation [SD]). The reads were delineated into 1,457 amplicon sequence variants (ASVs). Estimators of alpha diversity were calculated according to the Shannon index. Although no significant difference was detected among the groups, a trend toward decreased diversity was observed in the DSS-induced P2X7^+/+^ group (Figure 10*A*). The Venn diagram revealed that only 20 operational taxonomic units (OTUs) were preserved and shared among the groups, with greater superimposition observed among the P2X7^-/-^ mice (56%) (Figure 10*B*). Principal coordinate analysis (PCoA) revealed clustering of P2X7^−/−^ samples (blue and green dots) away from P2X7^+/+^ samples (red and orange dots). The P2X7^+/+^ controls were distributed unevenly, revealing heterogeneity within that group, which might have hindered a more comprehensive analysis (Figure 10*C*). The microbiota composition differed between the P2X7^+/+^ and P2X7^−/−^ controls, as well as between the DSS-induced and control mice (Figure 10*C* and *D*). The fecal microbiota was mainly composed of Bacteroidetes and Firmicutes across all groups, followed by Proteobacteria, Cyanobacteria, and Actinobacteria, among others (Figure 10*E* and *F*). Among the DSS-induced mice, the P2X7+/+ samples showed a greater relative increase in Proteobacteria abundance than the P2X7^+/+^ samples. In contrast, the abundance of the genus *Lactobacillus* (Firmicutes phylum) was relatively lower in the DSS-exposed animals than in the control animals, especially in the DSS-induced P2X7^+/+^ mice (*P*=.0094) (Figure 11*A–C*), corroborating the findings before and after DSS induction (Figure 10*E* and *F*). Bacteria belonging to the *Desulfovibrionaceae* family (Thermodesulfobacteriota phylum), *Roseburia* and *Marvinbryantia* genera (Firmicutes phylum), and *Odoribacter* and *Prevotellaceae* genera (Bacteroidetes phylum) had lower relative abundances in the DSS-induced group, whereas their levels were consistently greater in the P2X7^-/-^ control group. In contrast, in the DSS-induced group, there was a relative increase in the abundances of the *Faecalibaculum* and *Turicibacter* genera and the *Lachnospiraceae* family (Firmicutes phylum), especially in the P2X7^+/+^ group (Figure 11*D*). Interestingly, the *Marvinbryantia* and *Prevotellaceae* genera were exclusively detected among the P2X7^-/-^ controls, whereas the *Faecalibaculum* genus was detected only in the P2X7^+/+^ mice. Taken together, these results suggest that the intestinal microbiome plays a role in intestinal fibrosis, indicating that ATP-P2X7-mediated fibrosis is dependent on the abundance of specific microbes.

## Discussion

In this study, we investigated whether purinergic signaling through the P2X7 receptor is involved in the development of intestinal fibrosis in IBD. In human intestinal samples, we demonstrated that increased P2X7 expression was associated with local myofibroblasts, a finding that was more pronounced in patients with CD. In isolated human fibroblasts, P2X7 can be upregulated by various stimuli, including ATP, LPS, and inflammatory mediators, leading to increased cell migration and the production of ECM components. In vivo, we showed that chemical P2X7 blockade substantially attenuated the development of fibrotic changes, whereas P2X7^−/−^ mice developed markedly less intestinal fibrosis. Moreover, we documented changes in the intestinal microbiota before and after the induction of chronic colitis, which may contribute to the fibrogenic process and support a potential role for the P2X7 gene in microbial control within the intestine.

The increased number of α-SMA-positive cells in the intestinal mucosa is consistent with the notion that P2X7 plays a role in activated myofibroblasts in chronic inflammatory processes. Our findings corroborate recent evidence indicating that P2X7 is involved in the fibrotic processes observed in other organs.^12^ A recent study confirming increased P2X7 expression in surgical resections from patients with CD paradoxically found that P2X7 deficiency exacerbated fibrosis in an experimental model.^19^ In contrast, the present study, which employed a similar model of chronic intestinal inflammation, demonstrated that P2X7 is crucial in determining the long-term changes underlying intestinal fibrosis. The opposing results regarding experimental colitis may be explained by subtle differences in experimental design, DSS concentrations, animal conditioning, and likely differences in intestinal microbiota, which were analyzed only in the current study. Nonetheless, the substantial fibroblast heterogeneity previously demonstrated in resection specimens of CD strictures must be considered.^20^ Moreover, the multiple intercellular interactions within the intestine, along with the dynamic changes, including epigenetic modifications, may significantly influence the progression of fibrosis in intestinal inflammation. In this context, DAMPs, including the ATP-P2X7 pathway, have emerged as potentially relevant factors in the complex equation underlying intestinal fibrogenesis in IBD.

The experimental model used in this study appears to reproduce the long-term changes observed in human IBD, in which repetitive inflammatory cycles result in a combination of acute and chronic abnormalities.^21^ Regarding the anticipated chronic modifications in the experimental model, images generated by the endoluminal ultrasound system, obtained simultaneously with the colonoscopic view, confirmed a notable increase in intestinal wall thickening and stiffness in DSS-induced wild-type mice. In IBD, increased collagen deposition is characterized by an imbalance between collagen synthesis and degradation, leading to excessive ECM deposition and thickening and stiffening of the intestinal wall.^17^^,^^22^^,^^23^ As reported in the literature, collagen production from explants has been used to investigate the activation state of ECM-producing myofibroblasts within the chronically inflamed mucosa.^24^^,^^25^ The increased deposition of ECM components in tissue sections and the increased secretion of collagen and procollagen in the explant culture supernatants of DSS-induced wild-type animals, but not in P2X7-deficient animals, corroborated the long-term fibrotic changes in the model. Although P2X7 genetic ablation significantly abrogated both collagen and procollagen secretion, chemical treatment with A740003 inhibited only procollagen secretion, suggesting that additional mechanisms downstream of P2X7 receptor signaling may be critical to intestinal fibrogenesis.

Nevertheless, the inflammatory changes observed via colonoscopy and histological analysis highlight the model’s overlapping acute and chronic changes. Consistent with inflammatory activity, we observed increased production of proinflammatory cytokines, expected with innate immunity and Th1-type immune responses, typically observed in chronically active human^26^ and experimental IBD.^27^ A previous study of human IBD also revealed increased concentrations of IL-1β and TNF-α in inflamed tissue, which were modulated by the ATP-P2X7 pathway.^15^ IL-1β was recently shown to stimulate collagen production in a dose-dependent manner in vitro^28^ and to help maintain fibroblast activation, thereby acting in concert with TGF-β1 to promote ECM production.^29^ In intestinal explants from DSS-induced wild-type mice, increased secretion of TGF-β1, a vital regulator of intestinal fibrogenesis,^30^ and IL-10, a critical regulatory cytokine, also supports the complex, simultaneous activation of acute and chronic regulatory mechanisms in the experimental model.

IL-1β, which is directly regulated by the ATP-P2X7 pathway, has been implicated in immediate and early innate immune responses to microbial constituents, the activation of the inflammasome,^31^ and the increased intestinal permeability associated with NF-κB activation.^32^ NF-κB, in turn, is activated in epithelial cells and mucosal macrophages, leading to the secretion of a broad range of cytokines, including TNF-α and IL-1β.^33^ Regarding fibrosis, a previous study demonstrated that NF-κB blockade counteracted the fibrogenic ECM changes in an experimental model of chronic intestinal inflammation.^34^ In addition to NF-κB, ERK, a MAPK, was also shown to contribute to fibrogenesis, acting as a downstream mediator of TGF-β1, which is necessary for inducing intestinal fibroblasts.^35^ Therefore, the overexpression of NF-κB and p-ERK observed in DSS-induced wild-type but not in P2X7-deficient chronic model mice suggests that purinergic signaling through P2X7 modulates the activation of intracellular signaling pathways involved in the intestinal inflammatory response.

The increased expression of NLRP3 and caspase-1 in tissue sections from DSS-induced wild-type mice is compatible with the activation of targets downstream of P2X7 in the current model. In fact, upon exposure to diverse stimuli, such as extracellular ATP,^36^ microbial constituents,^37^ and NF-κB and MAPK signaling,^38^ NLRP3 becomes activated, releasing active caspase-1 from the inflammasome complex. In other tissues, such as the liver and kidney, the NLRP3 inflammasome has also been shown to play a role in chronic inflammation and fibrosis induced by factors such as extracellular ATP.^39^ This finding is consistent with the notion that the NLRP3 inflammasome is a crucial regulator of intestinal homeostasis and that its abnormal activation can contribute to the pathogenesis and disease progression of both human and experimental IBD.^40^

The disruption of epithelial integrity observed in this study, with goblet cell depletion paralleled by increased apoptosis, is compatible with chronic mucosal injury in the model. Previous studies have demonstrated that injured epithelial cells can become a crucial source of myofibroblasts, exhibiting profibrotic and proinflammatory activity, thereby contributing to fibrogenesis through type 2 epithelial-to-mesenchymal transition (EMT) in various tissues.^41^ In addition to ECM components, the intestinal epithelium niche comprises mesenchymal cells that may secrete cytokines and express TLRs, thereby directly participating in innate immunity, depending on the microenvironmental context.^42^ In this study, the expression of the transcription factor PPAR-γ was substantially lower in colon samples from the DSS-induced P2X7R^+/+^ than in those from the DSS-induced P2X7R^-/-^ and control mice. This finding corroborates the idea that PPAR-γ signaling plays a critical role in intestinal homeostasis, protection against inflammation, regulation of innate antimicrobial immunity,^43^ and the prevention of inflammation-driven intestinal fibrosis.^44^

We hypothesize that the ATP-P2X7 pathway can act differently depending on the extracellular ATP concentration and the target cell. High concentrations of ATP originate from the superficial layers of the gut mucosa and may open large pores in exposed cells, allowing the release of inflammatory cytokines and leading to cell death. In these regions, tissue destruction prevails, characterized by erosions and ulcers, where inflammation and cell death, fueled by the ATP-P2X7 pathway, are predominantly observed in epithelial cells. In earlier studies from our group, HCT8 epithelial cells exposed to 2 mM extracellular ATP experienced cell death.^45^^,^^46^ In another study from our group, regulatory T cells (Tregs) were found to be more susceptible to P2X7-mediated cell death induced by ATP treatment at a concentration of 1 mM.^13^ Nevertheless, the current study demonstrates that fibroblasts resist cell death induced by extracellular ATP, even when exposed to 2 mM ATP and when LPS or IL-1β is added. In contrast, low concentrations of ATP, arriving at distant locations from inflammatory hotspots and possibly more pronounced in the lamina propria and submucosa, would induce the opening of cation channels, such as demonstrated in this study, resulting in noncytolytic effects, as previously observed for cell proliferation, chemotaxis, modulation of energy metabolism in fibroblasts^47^ and the promotion of EMT.^48^ Previous studies demonstrated that ATP can directly stimulate mononuclear cells, such as dendritic cells and macrophages, thereby amplifying the inflammatory response.^49^, ^50^, ^51^ Moreover, the P2X7 receptor has been regarded as critical to determining how antigens are presented and how T cells are activated.^52^ Nevertheless, as demonstrated by the current study, ATP-P2X7 stimulation can also induce physiological changes in fibroblast activity, including cell migration and ECM synthesis. Therefore, in the lamina propria, the ATP-P2X7 pathway may also predominantly promote noncytolytic effects, contributing to a microenvironment that sustains inflammation and fuels tissue remodeling.

The increased relative abundance of Proteobacteria and the reduction in *Lactobacillus* abundance in the DSS-induced model, particularly in P2X7^+/+^ mice, are compatible with a microbial proinflammatory profile and consistent with previous reports in DSS-induced colitis and DSS-colitis-associated fibrosis.^53^ Bacteria also represent a source of extracellular ATP, and the ability to secrete ATP is particularly elevated among alpha- and gamma-proteobacteria.^54^ Therefore, in this study, the increased relative abundance of Proteobacteria observed in mice that developed chronic inflammation and fibrosis (DSS-induced P2X7^+/+^ mice) could represent an additional source of ATP, thereby enhancing the effects of ATP derived from tissue damage. Consistent with our findings, earlier studies revealed that increases in *Turicibacter* and *Lachnospiraceae* were also associated with the development of DSS-induced colitis.^55^ In contrast, *Faecalibaculum* was related to inflammatory and fibrotic changes in rats with colitis induced by 2,4,6-trinitrobenzene sulfonic acid (TNBS).^56^ The present study also revealed a reduction in potentially beneficial bacteria, including *Roseburia, Prevotellaceae, and Odoribacter,* consistent with previous observations in acute^55^ and chronic DSS exposure models.^55^ Notably, while the relative abundances of *Roseburia* and *Odoribacter* were increased in P2X7^-/-^ mice, *Marvinbryantia,* also regarded as a beneficial microorganism,^57^ and *Prevotellaceae* were exclusively detected among the P2X7^-/-^ controls. To our knowledge, we demonstrate for the first time a trend toward greater relative abundance of several protective bacteria in P2X7^-/-^ samples, supporting a possible role in preventing intestinal inflammation and fibrosis. The findings presented here, which revealed distinctive effects of the chemical insult and genetic background on the development of intestinal fibrosis, appear to be consistent with human studies highlighting the association between intestinal dysbiosis and clinically relevant gene expression profiles in patients with IBD.^58^ Nevertheless, given the small sample size and limited restoration with the P2X7 antagonist, it is difficult to attribute a causal role to microbial abnormalities in fibrosis. Still, this factor could represent a secondary phenomenon in the model.

The loss of epithelial elements observed in this study, including mucous-producing cells, further compromised the epithelial barrier, increasing mucosal exposure to the intestinal microbiota. In addition to triggering an immune response and driving inflammation, the intestinal microbiota has also been implicated in the development of IBD-associated fibrosis.^59^ For example, in a study of newly diagnosed pediatric CD, a risk-stratification model identified dysbiosis as a contributing factor in the development of strictures.^60^ In vitro, direct activation of primary human intestinal fibroblasts, characterized by increased collagen production, was demonstrated upon LPS stimulation.^61^ In experimental models of IBD, genetically engineered animals do not develop intestinal fibrosis when kept under germ-free conditions.^62^^,^^63^ Additionally, antibiotic treatment significantly improved the survival rate and reduced colitis and fibrosis in a mouse model of radiation-induced intestinal injury.^64^ Nevertheless, specific bacteria can directly induce a profibrogenic response in the gastrointestinal tract. For example, oral administration of live *Salmonella enterica* serovar Typhimurium has been used to induce intestinal fibrosis, and the effect has been shown to depend on both the mouse genetic background^65^ and the bacterial strain.^66^ Adherent-invasive *Escherichia coli* (AIEC), frequently isolated from the ileal lesions of patients with CD,^67^, ^68^, ^69^ induced fibrosis in mice after exogenous inoculation of AIEC strains NRG857c^70^ and LF82.^71^ As expected, we identified microbial dissimilarity between DSS-induced and control mice (ie, between inflamed and noninflamed mice) and between P2X7^+/+^ and P2X7^−/−^ mice, as observed in previous studies from our group.^14^^,^^72^

Although the current investigation revealed a clear association between the ATP/P2X7 pathway and the development of intestinal fibrosis, several important limitations should be acknowledged. First, the human studies are predominantly descriptive, and the in vitro analyses used both colon and skin fibroblasts. In in vivo studies, the number of experiments and the number of animals per experiment were relatively small, primarily due to technical difficulties in long-term animal follow-up. Moreover, the DSS-induced colitis model has several potential caveats, including variability in disease severity among animals, inconsistent chemical formulations, and differences in the inflammatory response, which do not fully replicate all aspects of human disease. Further studies investigating the role of P2X7 in chronic intestinal inflammation should also consider the use of additional antagonists and dosages, distinct experimental animal models and protocols, and the analysis of serial samples to assess the timing of changes that may occur during the process, while accounting for the parameters evaluated. Finally, antibiotics could provide valuable insights into the mechanistic role of the microbiota in the model and possibly substantiate a synergistic effect with the ATP/P2X7 pathway, contributing to the full development of chronic inflammatory changes and fibrosis. Additionally, this study did not investigate the microbiota as a potential source of ATP. In a previous study, ATP released from the microbiota was shown to modulate T follicular helper cells in the Peyer’s patches and to increase secretory IgA release.^73^ Moreover, in this study, we did not investigate the role of ectonucleotidases, which have been shown to regulate luminal ATP levels^74^^,^^75^ and to affect the development of inflammation in DSS-induced colitis.^76^ Nevertheless, the results were largely consistent and revealed several relevant differences, providing at least a solid foundation for future investigations.

## Conclusion

The current findings support the involvement of the ATP-P2X7 pathway in maintaining a local inflammatory milieu that supports the development of intestinal fibrosis. The regulatory mechanisms activated downstream of the P2X7 receptor, in combination with signals from an abnormal microbiota, promote the inflammatory response by converging intracellular signaling pathways and the inflammasome in a complex network of interacting molecules that form a stress-inflammation amplification loop, ultimately leading to fibrogenesis. In particular, ATP-P2X7 signaling induces calcium influx, fibroblast migration, and enhances ECM synthesis. Moreover, these mechanisms, orchestrated by P2X7 activation, disrupt the epithelial barrier and PPAR-γ signaling, further contributing to a pro-fibrotic microenvironment. Hence, we postulate that targeting P2X7 in IBD could be a novel approach to prevent chronic inflammation and tissue damage, potentially inhibiting excessive fibrogenesis.

## Materials and Methods

### Patients With IBD and Mucosal Specimens

#### Ethics statement

The Ethical Committee of the University Hospital of the Federal University of Rio de Janeiro approved the study protocol (approval ID: 188/08), which was implemented in accordance with the ethical standards outlined in the 1964 Declaration of Helsinki. All enrolled patients provided written informed consent before participating in the study.

#### Study population

Consecutive adult patients over 18 years of age who were regularly followed up at the outpatient unit for Intestinal Diseases of the Division of Gastroenterology at the University Hospital of the Federal University of Rio de Janeiro, a tertiary referral center, were enrolled in this study. The diagnosis of IBD was based on routine clinical, imaging, endoscopic, and histological parameters.

Demographics and clinical information, including age, sex, age at diagnosis, smoking status, presence of extraintestinal manifestations, disease extension (Montreal classification),^77^ and medical therapy at the time of enrollment, were obtained from hospital electronic medical records and patient interviews. The exclusion criteria consisted of patients receiving concomitant nonsteroidal anti-inflammatory drugs, patients with a diagnosis of unclassified IBD, patients with a history of previous cancer or acute or chronic enteric infection (eg, *Clostridium difficile*), and pregnant patients. All patients with IBD had active disease, as determined by the Harvey–Bradshaw Activity Index^78^ and the Simple Clinical Colitis Activity Index.^79^

The patients with CD were 4 women and 4 men, with a mean age of 38 years (range, 19–63 years). The mean duration of CD was 8 years (range, 1–15 years). The CD diagnosis was established in three-quarters of the patients before they were 40 years old. Three patients were treated with intravenous anti-TNF-α (10 mg/kg every 8 weeks) plus azathioprine (100–200 mg/day), 3 patients were treated with subcutaneous anti-TNF-α (40 mg/week), and 2 patients were treated with prednisone (10–40 mg/day) and azathioprine (100–200 mg/day). The disease involved both the ileum and the colon in all patients. None of the patients had proximal small bowel involvement. Regarding the predominant behavior of CD, 4 patients had the penetrating form, and 4 had the stricturing form. The patients with UC consisted of 4 women and 4 men, with a mean age of 44 years (range, 25–67 years). The mean duration of UC was 10 years (range, 2–22 years). Clinically, 4 patients had moderate-to-severe disease, and 4 had severe disease at the time of the study. Three patients were treated with anti-TNF-α (5 mg/kg every 8 weeks), 3 patients with azathioprine (100–200 mg/day), and 5 patients with prednisone (20–40 mg/day), azathioprine (100–150 mg/day), or mesalamine (2.4–4 g/day). Four patients had pancolitis, and 4 patients had limited left-sided colitis. The IBD samples from the patients with CD and UC were inflamed colon samples with relatively active disease and similar degrees of histological inflammation. The control group consisted of 3 men and 5 women, with a mean age of 41 years (range, 24–62 years): 3 with diverticular disease and 5 with benign polyps. Patients underwent colonoscopy, and tissues were obtained from at least 10 cm from the diverticula or polyps in macroscopically noninflamed areas. All mucosal specimens of the control patients were histologically normal. None of the control patients took any medication during the study.

### Experimental Chronic Colitis and P2X7^-/-^ Mice

#### Ethics statement

The institutional animal care committee of the Health Sciences Centre of the Federal University of Rio de Janeiro approved the use and care of the animals and the procedures reported in this study (approval ID: 059/20), following the guidelines of the Animal Research: Reporting of In Vivo Experiments (ARRIVE) [www.nc3rs.org.uk/ARRIVE (accessed on January 10, 2024)], developed by the National Centre for the Replacement, Refinement, and Reduction of Animals in Research (NC3Rs).

#### Experimental design and P2X7 blockade

To reduce heterogeneity in gut microbiota composition, we housed the animals in each treatment group in the same rack-mounted wire cage, with sufficient space to accommodate all the mice.^80^ The number of animals was calculated via the free software G∗ Power, with a 2-way analysis of variance (ANOVA) with an input considering Cohen’s F = 0.3; type I error = 0.05; power = 0.8; and numerator df = 80.

All the mice were randomly assigned to experimental group cages and subjected to a 4-week acclimatization period, during which they were dewormed twice with ivermectin.

DSS exposure damages the epithelial barrier, exposing deeper layers of the gastrointestinal tract to intraluminal antigens and triggering an inflammatory response similar to that observed in inflammatory bowel conditions. Through repeated cycles of DSS exposure, a chronic inflammation model can be obtained, allowing tissue regeneration during the pause periods. Water consumption was monitored and found to be similar among the groups.

Sixty age-matched, 6- to 8-week-old C57BL/6 male mice were used, with half being wild-type mice (P2X7^+/+^) and the other half P2X7-receptor-deficient mice (P2X7^-/-^). The animals were kept under specific pathogen-free conditions and continuously acclimated at 22°C under a 14/10-hour light/dark cycle. The mice received standard irradiated pelleted food and autoclaved water ad libitum. The P2X7 knockout mice were obtained from the Jackson Laboratory and bred at the Animal House of Transgenic Mice at the Federal University of Rio de Janeiro (UFRJ).

#### Experimental design

All mice were randomly assigned to experimental group cages and induced long-lasting chronic colitis by cyclical administration of 1% DSS (36,000–50,000 MW, MP Bioscience) in autoclaved drinking water. The induction protocol involved 3 cycles of 1-week DSS exposure followed by a 2-week break (7 days of DSS and 14 days of water). For the therapeutic protocol, P2X7^+/+^ mice received intraperitoneal injections of A-740003 (Tocris Bioscience), a P2X7-selective antagonist, 1 hour before the second and third DSS cycles. The mice were weighed weekly and euthanized between days 56 and 58 using carbon dioxide (CO_2_) inhalation.

#### Colonoscopy, endoscopic ultrasound, and distensibility assay

Regarding colonoscopy, briefly, mice were examined under isoflurane anesthesia at 1.5% in 1.5 L/min oxygen using a laboratory animal compact table Vevo anesthesia system (Visualsonics) and examined with a rigid video endoscope with an external diameter of 2.5 mm (Storz) assembled into a video camera for real-time evaluation of mucosal appearance and for recording images. eUBM images were acquired with Vevo 770 (Visualsonics) equipment with a center frequency of 40 MHz and the RMV704 probe (Visualsonics). This equipment generates ultrasound images with lateral and axial resolutions of 80 and 40 μm, respectively, at 34 frames/second. The combination of sonographic and optical images provides a transversal view of the colon wall during the inflammatory process or tumor development. The distal colon, 30 ± 5 mm from the anal verge, was examined in all experimental animals. Colon injury was scored by 2 independent observers using an adapted endoscopic index of colitis.^81^^,^^82^ Our previous experience with video colonoscopy using the eUBM system for experimental models of intestinal diseases^83^ enabled us to identify superficial and intramural changes in the present study.

Colon stiffness was assessed using a distensibility test with ultrasound, measuring the transverse section area 2 cm from the anus at 0, 1, and 2 minutes after administering 0.5 mL of saline solution (10 seconds after the start of saline administration).

### Euthanasia and Specimen Collection

After the colonoscopy and ultrasound image acquisition were performed, the animals were euthanized via asphyxiation with increasing concentrations of CO_2_ gas, followed by death confirmation through cervical dislocation. After euthanasia, the colon (corresponding to the region from the cecum to the distal portion) was removed and measured. The samples were then collected for histopathological analysis, cytokine and collagen measurements, and gene expression modulation analysis.

#### Histological analysis

The distal 2 cm of the colon was divided into portions for all experimental procedures. Colon samples were fixed in 40 g/L formaldehyde-saline (diluted in aqueous phosphate buffer, pH 7.3) immediately after removal from the mice. The samples were fixed for at least 24 hours, dehydrated in ethanol and xylene, and then embedded in paraffin. The paraffin blocks were cut into 5-μm-thick sections and mounted onto poly-L-lysine-coated slides, then stained with H&E using a Leica Microsystems, Ltd optical microscope equipped with a camera by 2 independent observers. The histological scores were evaluated for the presence and severity of ulceration, hyperplasia, and inflammatory infiltration by 2 independent observers. Ulceration was estimated based on the following scores: 0 = absent; 1 = focal superficial ulceration; 2 = diffuse deep ulceration; 3 = glandular disruption or deep ulceration; and 4 = diffuse glandular disruption or extensive deep ulceration. The following scores were used to estimate hyperplasia and cellular infiltration: 0 = absent; 1 = mild; 2 = moderate; 3 = severe. The total scores ranged from 0 to 10.

To further characterize the histopathological changes, we used the PAS technique. The density of goblet cells was determined as the percentage of PAS-positive cells within at least 500 epithelial cells in the crypts and the surface epithelium of longitudinally sectioned colonic pits. To evaluate the deposition of collagen fibers in the tissue, paraffin sections of colon samples were stained with phosphomolybdic acid-picrosirius red dye. Analyses were performed using a Leica Microsystems, Ltd optical microscope connected to a computer-assisted image analyzer.

#### Immunohistochemistry, double immunofluorescence, confocal laser-scanning microscopy, and assessment of apoptosis

Paraffin sections were cut onto slides pretreated with polylysine and subjected to indirect immunoperoxidase and double immunofluorescence techniques. Briefly, deparaffinized sections were incubated at 90°C in 0.01 M sodium citrate buffer (pH 6.0) for 30 minutes for antigen retrieval. Then, the slides were immersed in 3% hydrogen peroxide in methanol for 10 minutes to block endogenous peroxidase activity. After being rinsed in phosphate-buffered saline (PBS) containing 0.5% Tween 20 for 10 minutes, the tissue sections were incubated with 1.5% bovine serum albumin (BSA) + 0.1% Triton for 30 minutes and 5% BSA for 10 min. Immunological staining was performed using the following primary antibodies: rabbit polyclonal anti-caspase-1 antibody (1:500; ab1872); rabbit monoclonal anti-NLRP3 antibody (1:500; EPR23094-1) (both from Abcam); mouse monoclonal anti-p65 to NF-κB (1:200) and mouse monoclonal anti-p-ERK1/2 phosphorylated ERK (1:200) (both from Santa Cruz Biotechnology); and rabbit monoclonal anti-PPAR-γ antibody (1:200; C26H12, Cell Signaling Technology). Two sections from each sample were incubated with PBS alone and served as the negative controls. After overnight incubation in a humidified chamber at 4°C, the tissue sections were rinsed with PBS and incubated with EnVision FLEX/HRP detection reagent (SM802, Dako) for 30 minutes at room temperature. Development was performed with a solution containing hydrogen peroxide and diaminobenzidine (Dako). The samples were counterstained with Harris’s hematoxylin, dehydrated, and mounted onto slides using the water-free mounting medium Entellan (Merck Millipore). Image acquisition was performed via a computer-assisted image analyzer (Leica QWin Plus V 3.5.1, Leica Microsystems, Ltd).

In a double direct or indirect immunofluorescence study, the sections were incubated overnight at 4°C with a blocking buffer containing 2.5% BSA, 2.0% skim milk, and 8.0% fetal bovine serum (FBS), while shaking. The slides were rinsed once with PBS containing 0.05% Tween-20, then incubated with appropriately diluted primary antibodies in PBS. The tissue sections were incubated for 1 hour at room temperature with mouse monoclonal anti-α-SMA (1:200) or mouse monoclonal anti-vimentin (1:200) antibodies (both from Dako) and rabbit polyclonal anti-P2X7 (1:200) antibodies (Alomone Labs). After incubation, the slides were rinsed 3 times and incubated with Alexa 488-conjugated anti-rabbit IgG and Alexa 633-conjugated anti-mouse IgG (Molecular Probes) (1:1000) for 30 minutes at room temperature. Sections from each sample were incubated with either PBS alone or with a secondary antibody, serving as negative isotype controls. The slides were air-dried, fixed for 5 minutes in 1% paraformaldehyde, and mounted in an antifading medium containing 4′,6-diamidino-2-phenylindole (DAPI) (Vector Labs). The localization and fluorescence intensity of the proteins were observed with a Leica TCS-SP5 AOBS confocal laser-scanning microscope (Leica), which was used to capture representative images of each sample.

Apoptosis was assessed in histological sections via a TUNEL assay. Distal colon sections were stained via an ApopTag Peroxidase in situ Apoptosis Detection Kit (Millipore Corporation). First, the paraffin sections were deparaffinized, hydrated, and incubated with proteinase K. Endogenous peroxidase activity was blocked by immersing the sample in 3% hydrogen peroxide for 10 minutes. The slides were then rinsed in PBS and incubated with a TdT enzyme solution. The second sections of each sample were incubated without the TdT enzyme to serve as negative controls. We pretreated samples with DNase I (Sigma-Aldrich) as positive controls. Following the TdT enzyme step, the tissues were incubated with an anti-digoxigenin antibody conjugated to peroxidase. As described above, the sections were developed with a solution containing hydrogen peroxide and diaminobenzidine, counterstained with Harris’s hematoxylin, dehydrated, and mounted in a mounting medium. Apoptotic cells were defined as morphologically preserved TUNEL-positive cells and apoptotic bodies.

#### Quantitative assessment of colon sections

Quantitative analysis of tissue sections (under light microscopy) was performed using a computer-assisted image analyzer (Leica QWin Plus V 3.5.1, Leica Microsystems, Ltd). Any epithelial and lamina propria cells exhibiting identifiable reactivity distinct from the background were considered positive. The results of the quantitative analysis of the cell subsets are expressed as the number of cells per square millimeter of longitudinally sectioned colon tissue. Unaware of the experimental data, 2 independent observers examined all the tissue sections and captured the images.

#### Analysis of mRNA expression

Quantitative real-time polymerase chain reaction (qRT-PCR) was performed to assess the mRNA expression levels of genes involved in inflammatory and fibrotic responses. Following the manufacturer's protocol, total RNA was isolated from distal colon explants from mice via an SV Total RNA Isolation System (Promega). Total RNA was isolated from distal colon explants. The RNA samples were quantified and their purity determined using a Nanodrop 2000 UV–Vis Spectrophotometer (Thermo Fisher Scientific). Equal amounts of total RNA were reverse transcribed via a high-capacity cDNA archive kit. Next, qRT-PCR was performed with an ABI Prism 7500 (Applied Biosystems) and a CustomTaqMan R Array Gene Signature Plate (Thermo Scientific), which included the following genes for mice: *IL-4*, *IL-17A*, *IFN-γ*, *IL-1β*, *IL-22*, *TGF-β1*, *MMP3*, *MMP9*, *HMOX1*, *NOS2*, *CCR5*, *CC3CR1*, *NF-κB*, and *MAPK14*. The PCR cycles were performed according to the manufacturer’s instructions. The expression levels of the control housekeeping genes 18S and glyceraldehyde-3-phosphate dehydrogenase (GAPDH) were used to normalize the results. For data analysis, the ΔΔCt method was used to determine the fold change of all target genes in each sample with 95% confidence. qRT-PCR for each gene was performed in duplicate, and each experiment was repeated at least 3 times.

#### Assessment of ECM components in the colon

Colon samples were fixed in 40 g/L formaldehyde saline (diluted in aqueous phosphate buffer pH 7.3), embedded in paraffin, and cut into 5-mm-thick sections. Collagen fibers were stained with phosphomolybdic acid and picrosirius red dye. Alcian blue (pH 2.5) was used to stain sulfated and carboxylated acid mucopolysaccharides and sulfated and carboxylated sialomucins (glycoproteins). At least 5 different areas per tissue were evaluated. Analyses were performed using a Leica Microsystems, Ltd optical microscope connected to a computer-assisted image analyzer.

#### Cytokine, collagen, and procollagen measurements

Colon explants were cultured in Dulbecco’s Modified Eagle Medium (DMEM) supplemented with 10% FBS (Gibco-Invitrogen), 2 mM L-glutamine, penicillin (10,000 units/mL), and streptomycin (10 mg/mL) (all from Sigma Chemical Co) for 24 hours at 37°C in a 5% CO_2_ humidified incubator. The samples were centrifuged, and the supernatants were aliquoted and stored at −80°C. The explants were homogenized in M-PER medium, aliquoted, and stored at −80°C. Explant supernatants and homogenates were then subjected to cytokine and collagen measurements. To measure cytokine levels, we used a Cytometric Bead Array Mouse Th1/Th2/Th17 Cytokine Kit (BD Biosciences) with a FACSCalibur flow cytometer (BD Biosciences). The results were obtained and analyzed via BD CBA Analysis software. IL-1β and TGF-β were measured independently using individual ELISA kits (R&D Systems). For measuring collagen, we used BioVision’s Collagen Assay Kit (Cambridge Bioscience), a colorimetric assay. For measuring procollagen, we used Takara’s Procollagen Type I C-Peptide (PIP) enzyme immunoassay kit (Takara Bio). The total protein content of the biopsy samples was estimated using a Pierce BCA Protein Assay Kit (Thermo Fisher Scientific), which was used to normalize the results.

#### Microbiome composition

Gene-based microbiome profiling of fecal samples with taxonomic assignment was performed using high-throughput sequencing of 16S rRNA V3-V4 amplicons on the Illumina MiSeq platform (Illumina). Feces from each mouse were collected before starting the protocol and just before euthanasia and stored at −80 °C after snap freezing in liquid nitrogen.

Fecal genomic DNA was extracted with the PureLink Microbiome DNA Purification Kit (Thermo Fisher Scientific) according to the manufacturer’s instructions. The quality and quantity of the DNA were evaluated via a Nanodrop 2000 UV‒vis Spectrophotometer (Thermo Scientific). Fecal samples were analyzed for their microbiome using metabarcoding based on the 16S rRNA and V3-V4 regions.^84^ Libraries were prepared using the Nextera XT DNA Library Preparation Kit (Illumina) according to the manufacturer’s protocol. The quality of the libraries was determined via a 2100 Bioanalyzer instrument. A pool was made with an aliquot of each sample. The samples were sequenced via the MiSeq System (Illumina) and demultiplexed via the Illumina system.

As previously described, the quality of the sequences was verified with MultiQC, and the samples were analyzed with QIIME 2 (2022.8).^14^ The samples were denoised using the deblur extension. All ASVs were aligned using MAFFT,^85^ and the phylogeny was constructed with FastTree.^86^ Taxonomic annotation was performed using vsearch and the Silva v.123 database.^87^ Bar plots were constructed via barplot extension, and the alpha diversity was calculated based on Faith’s phylogenetic diversity^88^ via alpha-group significance (extension), and the beta diversity was calculated based on weighted UniFrac^89^ via beta-group significance extension. A Venn diagram was generated via InteractiVeen.^90^

#### Cells and in vitro studies

Human fibroblasts, derived from normal, non-malignant colon tissue, CCD-18Co cells (ATCC, CRL-1459), and human skin fibroblasts, HFF-1 cells (ATCC, SCRC-1041), were obtained from the American Type Culture Collection. Cells were treated upon exposure to IL-1β (10 ng/mL), LPS (1 μg/mL), TGF-β (5 ng/mL), and ATP (0.5 mM and 2 mM) or BzATP (100 μM) (all from Sigma-Aldrich), and analyzed to determine the modulation of P2X7 expression, and the effects of ATP-P2X7 pathway activation on calcium influx, cell migration, cell viability, caspase-3 activity, and the modulation of genes related to fibrogenesis.

Fibroblasts (passages 30‒34) were maintained in DMEM supplemented with 10% heat-inactivated FBS (Gibco-Invitrogen), 100 U/mL penicillin, and 100 mg/mL streptomycin (Sigma Chemical Co) at 37°C in a humidified atmosphere with 5% CO_2_. The migration of the fibroblasts exposed to BzATP, LPS, IL-1β, TGF-β, and the P2X7 selective antagonist KN62 (1 μM) (Calbiochem-EMD Biosciences, Inc), with or without the addition of RO-3306 (5 μM), a Cdk1 inhibitor (from Sigma-Aldrich) 24h before exposures, was assessed via a wound-scratch assay, followed by microscopic imaging. Briefly, CCD-18Co cells were plated at a density of 1 × 10^5^ cells per well in a 12-well plate. The following day, the supernatant was removed, and the wound-healing migration assay was performed by scratching a 200 μL pipette tip across each well. The wells were washed with incomplete DMEM, and the cells were treated for zero or 24 hours, after which photographs were taken.

P2X7 protein expression was analyzed via indirect immunofluorescence via confocal microscopy. For immunofluorescence, CCD-18Co cells were plated at a density of 2 × 10^5^ cells per well in 24-well plates. After reaching confluence, the cells were pretreated with KN62 for 1 hour or LPS for 24 hours, followed by ATP for 2 hours. After this procedure, the cells were fixed with 4% paraformaldehyde for 15 minutes at room temperature. The cells were washed with PBS for 10 minutes, permeabilized with 0.1% Triton X-100, and then incubated with 5% BSA for 10 minutes at room temperature. The cells were subsequently permeabilized twice with 0.1% Triton X-100 before incubation with a primary antibody against P2X7 (APR-008, Alomone) at a 1:500 ratio in PBS with 1.5% BSA overnight. The following day, the cells were washed, and an Alexa Fluor 488-conjugated goat anti-rabbit secondary antibody (1:500; Thermo) in PBS containing 5% BSA was added and incubated at room temperature for 1 hour. Finally, the wells were incubated with DAPI and washed 3 times with PBS. Fluorescent signals were detected via a fluorescence microscope (Cell Discoverer, Carl Zeiss).

Another valuable tool for evaluating P2X7R function is the assessment of changes in intracellular calcium ([Ca^2+^]) in CCD-18Co cells, as Ca^2+^ influx serves as a signal for multiple cellular processes, including immune responses, apoptosis, and inflammation.^91^^,^^92^ To assess Ca^2+^ influx in CCD-18Co cells, we plated cells at 1 × 10^5^ cells per well in a 12-well plate on 15-mm coverslips. In an initial set of experiments, we investigated whether ATP would activate P2X7R and whether LPS would enhance this activation. In this sense, some coverslips received 1 μM LPS 24 hours and 1 hour before the calcium assay. In another set of experiments, we tested whether A740003 would inhibit P2X7R activation. Briefly, cells in culture were loaded for 40 minutes with 5μM fura-2/AM (Molecular Probes), 0.02% Pluronic F-127 (Molecular Probes), and 1 mM probenecid (P8761, Sigma-Aldrich) in fluorimetry solution (145 mM NaCl, 5 mM KCl, 1 mM MgCl_2_, 1 mM CaCl_2_, 10 mM glucose, 5 mM 4-(2-hydroxyethyl)-1-piperazineethanesulfonic acid [HEPES], pH = 7.4) in an incubator with 5% CO_2_ at 37°C. After this period, the coverslips were mounted in an RC-20 chamber on a P-5 platform (Warner Instruments) for rapid perfusion on the stage of an inverted fluorescence microscope (Eclipse Ti-U; Nikon). Cells were continuously perfused with a fluorimetry solution and stimulated with 2 mM ATP (A2383, Sigma-Aldrich) or 100nM A740003 (3701, TOCRIS Bioscience). The variations in Ca^2+^ were evaluated by quantifying the ratio of fluorescence emitted by Fura-2 at 510 nm following alternate excitation at 340 and 380 nm, using a Lambda DG4 illumination system (Sutter Instrument), a 20× objective, a 510 nm band-pass filter (Semrock), and an Evolve 16-bit cooled EMCCD camera (Photometrics). Acquired images were processed using MetaFluor software (Molecular Devices). The time courses of the fura-2 fluorescence ratio for individual cells were analyzed in groups of 20 to 40 cells.

For RT‒PCR, HFF-1 cells plated at 5 × 10^4^ cells per well in a 12-well plate were pretreated for 24 hours with TGF-β, IL-1β, or LPS, or with KN-62, for 1 hour. After pretreatment, the samples were incubated with ATP for 2 hours and 30 min. Total RNA was isolated from at least 3 independent experiments using TRIzol Reagent (Invitrogen) according to the manufacturer’s instructions. After total extraction, a Nanodrop 2000 spectrophotometer was used to analyze the RNA concentration and purity of each sample. cDNA was subsequently synthesized via a high-capacity cDNA reverse transcription kit (Applied Biosystems). Finally, *COL1A1*, *ACTA2*, and *PPAR-γ* mRNA expression was analyzed via qRT-PCR with SYBR Green (Applied Biosystems). The primers used are listed in Table 1. An ABI Prism 7500 system (Applied Biosystems) was used to quantify changes in the mRNA levels. qRT-PCR was performed in duplicate. Gene expression analysis was conducted via the relative quantification formula and the comparative Ct (threshold cycle) method. For comparison of the samples via the creation of a delta Ct value, all quantifications were normalized by the expression of the constitutive gene β-actin, and the gene expression value of each target studied was obtained via the formula 2-ΔΔCt.

**Table 1:** Summary Information on the Primers Utilized

Primer	Sense	Sequence (5′–3′)	Length, *bp*	Reference
*COL 1A1*	Forward	TCTGCGACAACGGCAAGGTG	146	93
*COL 1A1*	Reverse	GACGCCGGTGGTTTCTTGGT	146	
*ACTA2*	Forward	TCAATGTCCCAGCCATGTAT	80	94
*ACTA2*	Reverse	CAGCACGATGCCAGTTGT	80	
*PPAR y*	Forward	AAAGAAGCCGACACTAAACC	150	95
*PPAR y*	Reverse	CTTCCATTACGGAGAGATCC	150	

For cell proliferation assessment, we used the BrdU Cell Proliferation Assay Kit (Chemicon International). Cells were rinsed with PBS, trypsinized, counted, and seeded at a density of 2 × 10^5^ cells/mL in 96-well plates, with 100 μL per well. After overnight attachment, cells were treated for 24 hours in triplicate with different reagents, including BzATP, LPS, IL-1β, TGF-β, and KN62. BrdU was added to the wells 4 hours before the plate reading at 450 nm.

To assess cell viability, parallel experiments with the same settings were performed. However, instead of BzATP, we treated cells with ATP at 0.5 mM and 2 mM. The labeling reagent MTT (Sigma-Aldrich) was added to wells, and the plates were read at a wavelength of 595 nm. The MTT test serves as an indirect marker of proliferation and cell viability by measuring mitochondrial activity.

For analyzing caspase-3 activity, HFF-1 cells were incubated at 2 × 10^5^ cells per well in a 24-well plate at 37^o^C in a 5% CO_2_ humidified incubator in DMEM (Gibco/Invitrogen) buffered with 3.7 g/L sodium bicarbonate and 5 g/L HEPES (Sigma-Aldrich) and supplemented with 100 U/mL penicillin, 100 lg/mL streptomycin (Gibco/Invitrogen), 1% L-glutamine (Sigma-Aldrich), and heat-inactivated 10% FBS (LGC Bio). Cells were then treated with ATP at 0.5 mM and 2 mM, LPS, IL-1β, TGF-β, KN62, and Triton X-100 to induce cell death or with vehicle control (dimethyl sulfoxide [DMSO], 0.2%). All treatments were performed in duplicate. After 24 hours, cells were harvested, washed twice in cold PBS, and resuspended in chilled lysis buffer. The cytosolic extracts were then subjected to caspase-3 activity measurement according to the manufacturer’s instructions (Caspase-3 Colorimetric Activity Assay Kit, Chemicon Millipore).

### Statistical Analysis

Significant differences between the experimental groups were identified with the paired-samples *t*-test, Wilcoxon matched-pairs signed-rank test, or Welch’s ANOVA test, in which multiple comparisons were carried out via Dunnett’s T3 test, as appropriate. Individual values are presented, along with their means and SDs. The survival data are presented as Kaplan-Meier survival curves and were analyzed with the log-rank test. All tests were 2-tailed, and statistical significance was defined as *P* values less than .05.
